# Antioxidants and Bioactive Compounds in Food: Critical Review of Issues and Prospects †

**DOI:** 10.3390/antiox11040742

**Published:** 2022-04-08

**Authors:** Mia Kurek, Nadjet Benaida-Debbache, Ivona Elez Garofulić, Kata Galić, Sylvie Avallone, Andrée Voilley, Yves Waché

**Affiliations:** 1 Department of Food Engineering, Faculty of Food Technology and Biotechnology, Pierottijeva 6, 10000 Zagreb, Croatia; ielez@pbf.hr (I.E.G.); kgalic@pbf.hr (K.G.); 2 Laboratoire de Biochimie Appliquée, Faculté des Sciences de la Nature et de la Vie, Université de Bejaia, Bejaia 06000, Algeria; nadjet.benaida@univ-bejaia.dz; 3 QualiSud, University of Montpellier, 34000 Montpellier, France; sylvie.avallone@supagro.fr; 4 CIRAD, Institut Universitaire de Technologie d’Avignon, 84029 Avignon, France; 5 International Joint Research Laboratory “Tropical Bioresources & Biotechnology” UMR PAM, Institut Agro Dijon, Université de Bourgogne, 1 Esplanade Erasme, 21078 Dijon, France; andree.voilley@u-bourgogne.fr (A.V.); yves.wache@agrosupdijon.fr (Y.W.); 6 The School of Biotechnology and Food Technology, Hanoi University of Science and Technology, Hanoi 100000, Vietnam

**Keywords:** bioactive compounds, encapsulation, food grade, bioavailability

## Abstract

This review paper gives an insight into the effective delivery mechanisms for health-promoting substances and highlights the challenges of using antioxidants and bioactives in foods. The selection criteria for choosing bioactives and their extraction in bioavailable form with their adequate incorporation techniques and delivery mechanisms are covered. Moreover, an overview of existing methods for determination of bioactivity is given. The importance of scientifically evaluating the effects of foods or food components on consumer health before making claims about the healthiness is aligned. Finally, a scientific perspective on how to respond to the booming demand for health-promoting products is given, and we acknowledge that despite the work done, there are still many challenges that need to be overcome.

## 1. Introduction

After a long period from the birth of mankind to recent times, in which the main objective of agriculture and food was to feed people, in recent years the objective has evolved to make people healthier by increasing life expectancy. This can be seen in the strategies of large companies such as Nestlé, Danone, and many others, which have transformed themselves from food companies to nutrition and health companies, as can be seen in their mottos and mission statements that talk about health or life. As a result, many food manufacturers are seeking to make health claims for their products, making health and nutrition communications a central part of their marketing strategy and launching initiatives to help address human health challenges. These challenges include the control of effective delivery mechanisms in addition to the identification of health-promoting substances. In this development, food authorities and scientists have a great responsibility to help consumers adopt sustainable and healthy behaviors and not fall into the commercial traps posed by supposedly healthy products that have no bioactivity or, worse, negative effects. 

**Figure 1:**
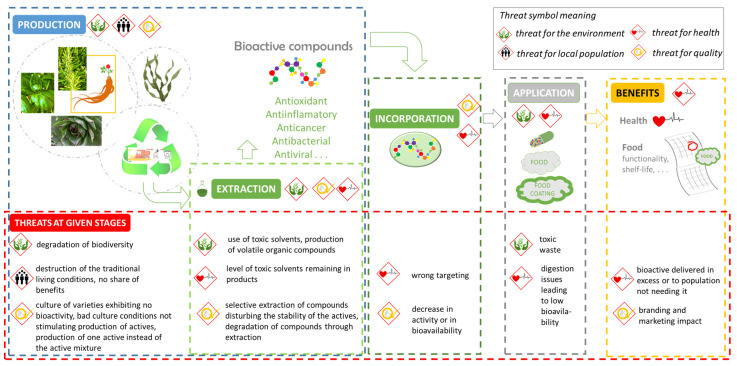
From raw material to the meeting of consumers’ needs.

In this context, many questions arise regarding the quality of bioactivity from agricultural products to food and consumers (Figure 1 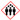, 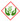, 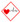, 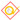). The purpose of this review paper is to address these issues and highlight the challenges of using antioxidants and bioactives in foods (Figure 1). Issues have been selected from the expertise of the authors, discussions carried out at L’Institut Agro Dijon with worldwide experts, and published reviews and lectures. Examples illustrating these issues are proposed by authors from their own experience.

## 2. Ways of Production of Bioactives and Impact on Their Quality, on the Biodiversity, Environment, and on Human Populations

The global economy is so hungry for healthy products that many companies that make food, from small start-ups to the largest multinationals, dream of finding the right product that will allow them to have nutritional claims on their product. However, many companies do not have the expertise to find the right product. When visiting the largest food fair in the world, the Salon International de l’Alimentation (SIAL), year after year, it is amazing to see that in some countries almost all exhibitors present the same plant extract. The search for new bioactives is a real problem, and looking at old herbal books and pharmacopoeias several centuries old, it seems that despite new technical and scientific possibilities to evaluate the activity, most of the active ingredients have been known for centuries [1]. Most attention is given to a small list of “superfruits/superplants” that are being advanced in agricultural development programs. However, the discovery of some new plant varieties with great bioactivity may shake up some communities or regions.

In the present communication era, the influence of marketing on consumer decisions is more than crucial and, unfortunately, sometimes leads to the challenge of “food for profit” instead of “food for life”. When communicating about bioactives that may be present in some plants, it sometimes seems as if the world goes crazy about a single plant. The first data are often collected on samples used in traditional medicine. This communication about the superpower of bioactives creates a demand from companies and/or consumers. As an initial impact, the harvest increases dramatically to change the scale from a traditional community’s pharmacopeia to supplying the world market with healthy products. If possible, the products are then cultivated. The history of Vietnamese ginseng (*Panax vietnamensis* Ha et Grushv., Sâm Ngoc Linh and varieties) illustrates this classic scheme. This plant has long been used in the traditional pharmacopeia of the Xedang ethnic minority living in the mountains of central Vietnam near the Laotian border, but was unknown outside this area for most of history. In 1973, it was discovered by people outside the ethnic minority and became very popular. About 10 years later, it received its name [2], and, according to *Le courrier du Vietnam* (published on 31 July 2011), was included in the International Union for Conservation of Nature’s (IUCN) Red List of endangered species only a decade later [1,3]. The International Union for Conservation of Nature’s Red List of Threatened Species was established in 1964 and has evolved to become the world’s most comprehensive source of information on the global status of animal, fungus and plant species threatened with extinction. It is a powerful tool for informing and catalyzing biodiversity conservation and policy changes, critical to protecting the natural resources that humanity needs to survive [4]. Fortunately, the *P. vietnamensis* Ha et Grushv. species has been conserved with great effort in the same primary forests where it was discovered [5]. The price offered for the plant for use as a bioactive or for cultivation purpose was so high that it was overexploited by the local population. Meanwhile, other highly valuable species that can grow only in the same mountain forests were cultivated in the same places (e.g., *Amomum tsaoko* Crevost et Lemaire/Tsao-Ko Cardamon [6]), creating competition among the Red List species culture in a place that was previously a primary forest [7]. 

When possible, crops are grown on accessible land, but because active compounds and especially antioxidants are produced by plants in response to stress, we must face the fact that sunlight, moisture, temperature, and especially temperature fluctuation often reduce the plant’s need for antioxidant compounds and thus its bioactivity [8]. With intensive agriculture, a plant can also be loaded with agricultural chemicals. It is usually considered that the benefits of eating plants counterbalance the effect of the phytochemicals contained in the plant. However, in the case of expensive superfruit preparation, this interest may be questioned. 

For high value-added plants, a hydroponic system can allow growers to cultivate them under perfect growing conditions and without pests [9,10]. For example, tropical ginseng can be cultivated in Belgium, which brings controlled health benefits [11,12]. In this case of cultivation for a company far from the origin of the plant, the Nagoya Protocol now guarantees benefit-sharing with the community living at the cradle of the plant, despite numerous gray areas in this protocol [13,14].

As intensive culture can reduce plant bioactivity, the use of microorganisms during culture for triggering or afterward, during a fermentation of the product can lead to higher bioactivity [15,16]. For example, elicitation by fungi has been used to produce glyceollin in soybeans [17]. These phytoalexins from soybeans have antibacterial, antifungal, and antinematode activity, as well as antiproliferative, antiestrogenic, anti-inflammatory, antioxidant, and anticholesterolemic activity. Therefore, they are being investigated for their medicinal properties against hormone-dependent cancers and metabolic and cardiac diseases [18]. They are formed by P450-mediated hydroxylation of 3,9-dihydroxypterocarpan following induction of this enzyme after elicitation by *Aspergillus* spp. [19]. Bioactive substances can also be produced by chemical or biotechnological synthesis. Although chemical synthesis generally has a bad image, biotechnological production can be interesting because it can be produced under the label of natural and organic production. Such production can also be sustainable if the compounds are produced from renewable crops, even more so if they are derived from waste or by-products [20]. If they are produced with a microbial producer hidden in the food (under the label “starter”), they can be suitable for a clean label strategy [21,22]. Unfortunately, it is often observed that a single compound does not have the same properties as the whole plant. A well-known example of bioactivity in flavor is the use of vanillin instead of vanilla. While vanillin brings the vanilla flavor to chocolate, vanilla enhances the overall flavor of chocolate, resulting in a complex flavor that is not dominated by vanilla flavors [23]. In some cases, the mechanisms of interaction between the bioactive and other components are known, such as the better stability of lycopene in plants compared to isolated lycopene, which can be explained by the protective role of other carotenoids in Gac (*Momordica cochinchinensis* Spreng.) [24,25]. 

Despite the drawbacks of using less stable or less effective antioxidants through strategies that attempt to control nature, some producers may be tempted to use genetically modified organisms (GMO) to produce an isolated active ingredient or to increase the production of active ingredients in the plant. Because perceptions of these strategies vary widely around the world (depending on the general perception of the populations about whether man can improve the nature through science or whether changes always bring catastrophic side effects) there are different perceptions about whether GMO-made products used for health or for food present an actual risk or not.

## 3. Extracting Bioactives

Plants are an inexhaustible source of bioactive compounds that have been used by men since ancient times as folk medicines [26] and as preservatives of food [27]. Medicinal plants have always been of interest to scientific research and to the chemical and pharmaceutical industries because of their multiple applications for their antioxidant [28], antibacterial [29], stimulative, and inhibitory properties [30,31].

Extraction is an important step in the itinerary of phytochemical processing for the discovery of bioactive constituents from plant materials. The extraction of primary and secondary metabolites from plants can be challenging because the inappropriate choice of extraction methods could alter or destroy the bioactive compound. Therefore, the discovery and extraction of bioactive compounds from natural sources is of growing interest to drug developers [32,33]. 

### 3.1. Basic Extraction Techniques

The basic extraction process includes steps such as collection, pre-washing, drying or freeze drying of plant materials, grinding to obtain a homogeneous sample, and often improving the kinetics of analytical extraction [34,35]. This section focuses on issues arising from the applications of several extraction techniques such as maceration, soxhlet, and hydrodistillation by using solvent extraction [36]. The fundamental principles for extracting bioactive compounds from natural plant materials, have also been reported. Namely, the methods and solvents considered diversely influence the extraction of molecules with different structures [37].

Solid-phase extraction (SPE) is the most widely used technique for the treatment of aqueous samples and extracts in the laboratory [38]. Generally, SPE methods require large amounts of organic solvents, high energy expenditure, and are time consuming. Aqueous-based methods are particularly promising because water is cheap and safe. However, these methods suffer from a reduction in extraction efficiency. The mass transfer rate of soluble ingredients into the solvent depends on the concentration of the ingredients. In addition, heating the solvent can also improve mass transfer because of better solubility [39,40]. Low extraction yields are due to the presence of polysaccharides such as hemicelluloses in the cell wall, time inefficiency, co-extraction of undesirable components, and the presence of trace organic solvents in the extract [41,42]. Another issue in the extraction of active compounds is their sensitivity to heat. Extraction is higher when the heating time [43] and temperature [44] increases. Unfortunately, for heat-sensitive compounds, the yield may be lower due to simultaneous degradation, although some examples have shown that moderate heat can improve the antioxidant properties of actives by changing the isomer content. However, authors suggest that extraction at low temperatures in pressurized microwave-assisted extraction mechanisms can attain reasonable extraction efficiency [45,46]. 

### 3.2. Environmentally Friendly Solvents

Recently, there is an increasing need for green and sustainable approaches that result in extracts with low environmental impact [46,47]. Great efforts are being made to make the extraction process more environmentally friendly and effective by using natural deep eutectic solvents (NADES) [46] and ionic liquids (ILs) [48]. Comparison of organic solvents with ionic liquids (ILs) was updated by Plechkova and Seddon (2008) [49]. Ionic liquids (ILs) are recognized as an environmentally friendly alternative to volatile organic solvents, especially in the fields of food, flavors, fragrances, and medicinal plants [50]. Studies reported the performance of ILs in the extraction of polyphenols from plant material, but the final separation of the active compounds was very difficult [50]. Additionally, switchable ILs have been developed that can be easily separated from the products [51,52]. Nevertheless, ILs are usually used only on a very small scale and are not compatible with industrial extraction. The biological effects of extracts from bioactive compounds obtained with NADES and ILs could change [51]. Therefore, researchers must be cautious when claiming that these alternative solvents have no toxicity and are safe for human consumption [53]. However, it is also possible to use non-toxic agricultural products to produce these ILs, and in this case, this technique is predicted to have a bright future [54].

### 3.3. Novel Extraction Techniques

New extraction methods have received a lot of attention in the last 10 years.

Assisting techniques or techniques using original fluids, which were previously considered as non-conventional methods, such as supercritical water, supercritical fluid, microwave-assisted extraction (MAE), ultrasound-assisted extraction (UAE), enzymatic assisted extraction (EAE), and supercritical fluid and pulse electric field (PEF) are becoming more popular and more efficient methods [51]. Moreover, ILs solutions in MAE of polyphenolic compounds from plant samples is a promising prospect [55]. Similarly, UAE is an environmentally friendly and rapidly developing technology suitable for upscaling and improving the extraction efficiency of bioactive compounds [56]. UAE showed the best performance, resulting in an extract with a phenolic content from grape skin twice as high and in a shorter time (9 min) than that obtained by mechanical agitation [57]. Comparison of mentioned novel and conventional extraction methods of polyphenols from plants are given in Table 1, and comparison of efficiency in the grape seed example in Table 2. 

Among the innovative green technologies, supercritical fluid extraction (SFE) is one of the most interesting because of the favorable properties of carbon dioxide (CO_2_) that, as a dense solvent, has a low critical temperature [68]. The image of supercritical CO_2_ is nice and allows manufacturers to put the “organic” label on their products, but the technique requires expensive equipment, especially for more specific extractions such as selective pressure relief fractionation. 

More recently, ultrasonic-assisted NADES combined with the macroporous resin enrichment method was reported as an exceptionally effective process and recyclable strategy for flavonoid extraction from *Acanthopanax senticosus* (Rupr. And Maxim.) [69]. 

The increased release of phenolic compounds by enzymes was previously described [70]. The application of an enzyme for extraction manages to release the phenolic compounds, making them easier to extract and improving their extraction rate, selectivity, and yield. Combination of enzymes (cellulase, pectinase, and tannase) increased the extraction yield up to 112% and showed three times less in comparison with the solvent extraction method [70]. In addition, the use of a complex enzyme or different enzymes has a strong effect on the outcome and allows the development of a customized extraction formula to obtain a value-added final product [71]. However, EAE has commercial and technical limitations. Enzymes are relatively expensive, cannot completely break down plant cell walls, and their behavior has been rigidly limited by environmental conditions [72] Recently, it has been reported that ultrasonic waves can improve the ability of enzyme extraction [73]. 

There are several reviews in the literature dealing with one or more extraction techniques [33,50,51]. Many new methods have been developed, but so far no single method is considered as standard for the extraction of bioactive compounds from plants. In particular, these authors have emphasized that the extraction method should be selected depending on the target compounds to be isolated. This implies understanding the nature of the plant samples [36] the critical input parameters [74], the sensitivity of the molecules of interest, and the enduse [75]. 

The application of the response surface method becomes a useful and valuable tool in the field of bioactive compound extraction [76]. Temperature, solvent concentration, solid–liquid ratio, microwave frequency, and extraction times are studied as independent variables to obtain the optimal conditions for extraction and maximize the bioactive compound content [77,78].

## 4. Protecting and Delivering Bioactives

In order to develop new food products, it is necessary to find a way to add functionality to the selected foods. Even though bioactive compounds initially have many advantages, they are often not stable in their original state and tend to degrade quickly. Not only are they sensitive to heat and light, but if not stored properly, they can influence the development of off-flavors and interact with other food components. Logically, active ingredients in foods must remain fully functional for as long as necessary. Therefore, they require appropriate strategies to maintain bioactivity at all stages of the product’s life, from manufacture to incorporation and storage to consumption, ingestion, and absorption during digestion in the gastrointestinal tract (GI) [79]. In response to the abovementioned issues, innovative encapsulation technologies have been proposed. 

The encapsulation process provides a way to stabilize sensitive bioactive compounds by appropriately structuring the systems. Bioactive material is coated with another single material or combination of materials (known as core and shell); e.g., polymeric carrier material, creating particles with different diameters. Encapsulation is used in different fields for different purposes, so core and shell materials for encapsulation vary depending on the application. A simple distinction can be made: (a) Core materials used in food may be aroma molecules (e.g., flavors) protected by a shell of gum arabic or dextrin and mixed directly into food formulations; whereas in the pharmaceutical field (b) common core materials are therapeutic substances (e.g., quercetin) enclosed in liposomes [80]. In the case of flavorings (case a), release may begin already before ingestion. Indeed, flavors are ingested by sniffing, and then their release continues in the mouth. In the mouth, in a complex process, flavor compounds are gradually released from the food matrix, mixed with saliva, and transferred to the pharynx, which then serves as a reservoir for further flavor release, but in this case the initial release action is complete [81]. In the example with quercetin (case b), about 90% of the ingested quercetin should be metabolized in the intestine to produce a therapeutic effect. Therefore, the shell material must protect this valuable bioactive compound throughout the gastrointestinal tract, which is exposed to a neutral to highly acidic pH medium. To address the above examples, in case a encapsulation of flavor compounds is used for the purpose of controlled release (premature, sustained, or alternate release, e.g., the release of flavors during a baking process), protection of ingredients (e.g., protection of flavors from oxidation, loss, and heat exposure), masking flavor (e.g., reducing the perception of bitterness of caffeine in breakfast bars), and enhancing flavor (e.g., heat (capsaicin) amplification) [82]. For the therapeutic compounds in case b, drugs and bioactive therapeutic lipid nanoparticles, polymeric nanoparticles, biopolymeric nanoparticles, magnetic nanoparticles, mesoporous silica nanoparticles, or cyclodextrins are intended for oral, intranasal, intravenous, or topical admission [80]. 

In the food sector, encapsulation is used for artificial sweeteners, acid bases, colorants, preservatives, antimicrobials, leavening agents, antioxidants, substances with undesirable flavors, odors and nutrients, in addition to the flavors already mentioned [83]. Encapsulated compounds benefit from the protective effect against degradation, volatilization or unwanted interactions with the matrix material, enhancement of compatibility between the biopolymer and the active substance, increase in availability and bioavailability, controlled release or/and stimulatory responsive release to prolong the activity of the active ingredient at a specific site in the body and to reduce changes in sensory properties of food or to comply with regulatory limits [84]. Microencapsulation can be used to protect sensitive cosmetic ingredients from harmful processes such as degradation by oxidation or polymerization during drying and/or thermofixation and garment storage. Cosmetotextiles are a unique combination of fabrics and incorporated skin care ingredients used, for example, as an anti-cellulite/slimming feature in hosiery, lingerie, and garments designed for good synergy between textile construction and cosmetic finishing [85]. 

The complexity of encapsulation systems is more than obvious. Delivery systems can be more or less sophisticated, depending on the physicochemical properties and structure of the bioactives, the structure and capacity of the carrier matrix, and the site of targeted delivery [86,87]. It would be extremely difficult to list all potential applications, so only an overview of some techniques is provided in this article. 

In the food sector, edible films and coatings represent an exceptional opportunity to transport bioactive substances, either to protect them and deliver them in their native form or to deliver them in a previously encapsulated form. These approaches were primarily aimed at extending the shelf life of foods, but they also impart important nutraceutical functions to foods and are beneficial to both food and consumers by reducing the risk of disease. An edible polymer matrix serves as a carrier for previously encapsulated bioactives. Recently, emulsions and nanoemulsions, core-shell nanofibers, cyclodextrins and liposomes have been preferred [84,88]. Their main advantages and disadvantages are given in Table 3.

### 4.1. Emulsion Based Structures

Emulsification is the process of dispersing at least two immiscible liquids, where one of the liquids is dispersed as small spherical droplets in the other to form a semi-stable colloidal mixture with polar and non-polar regions [89]. For example, a stable encapsulated fish oil-in-milk emulsion might be prepared in order to fortify cakes with omega-3 fatty acids to improve food dietary intake [90].

The dispersion of lipid droplets of nanometric size (50–200 nm) in an aqueous phase, leads to the formation of nanoemulsions [91,92]. These structures are being developed to facilitate the incorporation of hydrophobic bioactives into foods. For example, resveratrol was loaded into zein nanofibers for the fortification of apples [93] and whey protein isolate nanofibers with carvacrol were proposed as edible coatings for salted duck egg yolk [94]. The latest delivery technique generally used to deliver vitamins and drugs is known as liposomal encapuslation. Liposomes act as healing promoters to targeted body organs [95,96]. Interest in liposomal technology has rapidly increased because it can encapsulate both water-soluble and non-water-soluble molecules (such as vitamin C and vitamin B). Simple nanoemulsions are sometimes not sufficient in providing the desired functionality like delayed release or early degradation in the gastrointestinal tract; thus there are additional solutions proposed to extend the functional attributes of nanoemulsions. By trapping the small lipid droplets inside of biopolymer microgels the storage stability and releasing profile might be properly tailored. Commonly, nanoemulsions or lipid droplets are mixed with biopolymer solution and then they are altered to promote biopolymer gelation resulting in the formation of stable microgels. Microgels are prepared by coacervation which involves mixing two oppositely charged biopolymers (e.g., cationic protein and anionic polysaccharide) to obtain microgels with active droplets inside [97]. Coacervation is a process that occurs as a result of phase separation of the colloid-rich layer from the colloid-poor layer. The release of compounds from these systems depends on the stability of the coacervate and can be controlled by pH (similar to human GI tract), particle size, structure, loading value, degree of crosslinking, temperature, and dispersion medium [97,98]. The use of complex coacervation in gum arabic and gelatin for sweeteners (e.g., aspartame) and flavors in chewing gum is well known. For example, when pink pepper essential oil was microencapsulated by spray drying of single-layer emulsions, stabilized by soy protein isolate, and of double-layer emulsions, stabilized by soy protein isolate/high methoxyl pectin, microcapsules modulated release of volatiles, compared with pure essential oil and led to a reduction of microbial growth in whole milk and skim milk [99]. This example is evidence of the positive effect of microgels on the functionality of active compounds in food products. 

Solid lipid nanoparticles (SLNs) and nanostructured lipid carriers (NLCs) are structurally similar to nanoemulsions, but remain solid at room temperature because the lipid phase is either fully or partially crystallized. Crystallization of the lipid phase can improve the stability of encapsulated substances by slowing the diffusion of prooxidants [100]. This method is widely used in the pharmaceutical industry to deliver drugs for cancer therapies, antimicrobials, treatment of central nervous system diseases and/or disorders (other than cancer and infections), site-specific treatments, nanovehicles not intended for a specific therapeutic area (i.e., without an indication, including SLN for diagnostic purposes), and the remaining drugs for various conditions or diseases [101].

### 4.2. Nanofibers

Nanofibers consist of long thin fibrous materials, processed by a non-thermal electro-hydrodynamic process, known as electrospinning, in which charged polymers are subjected to a high electric potential [93,102]. Because encapsulation techniques whereby high temperature processing is required can often be detrimental to the active properties of many antimicrobial agents and antioxidants (e.g., essential oils EO), electrospinning is a proposed delivery vehicle in food nanotechnology. This is a method for production of ultrafine nanosize fibers by charging and ejecting a polymer melt or solution through a spinneret under a high-voltage electric field and to solidify or coagulate it to form a filament [103]. Authors [93] found that controlled release of active compound was achieved in vitro simulated gastrointestinal conditions when nanofibers were used. Sustained release with protected bioactivity of essential oils was recently reported for a double layer-chitosan-flaxseed mucilage-nanofibers [104].

### 4.3. Stabilisation of Active Compounds by Water Removal

Spray drying and freeze drying have the common objective of water removal and have attracted much attention for industrial applications. Removing water through drying provides numerous benefits with most important improved stability of labile high valuable bioactive molecules. Both processes are commonly employed as the final stage in the preparation of the inclusion complexes. From an economic point of view, powders have lower package/storage/transportation costs, while the storage stability of the associated bioactive compounds is improved [105,106,107]. 

Inclusion complexes are formed by entrapping the bioactive molecule in a cyclic oligosaccharide structure (e.g., α-cyclodextrin, β-cyclodextrin, and γ-cyclodextrin) [108]. Toroidal shape of the lipophilic cavity provides a suitable size and nonpolar environment for a noncovalent inclusion complex. This makes cyclodextrins a good host for lipophilic guest molecules even though they are water soluble. Therefore, they are suitable carriers for many phytochemicals known for their antioxidant, antifungal, anticancer, antiviral, anti-inflammatory, and antiphlogistic activities [109]. For example, by complexing herbal extracts with cyclodextrins, one can increase the bioavailability of undesirable substances such as pesticides or contaminants [110]. Recent studies also showed that cyclodextrins are not only carrier molecules, but are also digested and fermented in the digestive tract [111]. In particular, a promising effect on energy metabolism was demonstrated, making an interesting venue for further investigation of potential nutritional or pharmaceutical applications [110]. 

Spray drying is a well-known method for the preparation of microspheres and microcapsules for drug delivery. It involves the conversion of a liquid material into dried particles by using a gaseous hot drying medium [112]. This method is already widely used in the food industry, for example, to convert fruit juices into powder form. It is the preferred method for preserving the activity of many thermally sensitive materials which may require extremely consistent, fine particle size. The main disadvantages are still the high capital and overhead costs.

Freeze drying (lyophilization) is a process in which a completely frozen sample is dehydrated under vacuum. This process is based on the sublimation of the water present in a product, so the ice passes directly from a solid to a steam. This results in a reduction in water activity and consequently slower deterioration processes [113]. If a fine powdered form is required, then differently to spray drying, freeze drying requires an additional grinding or milling step. In the food industry, freeze drying is most commonly used to produce instant coffee, or dried fruits such as apples. Freeze-dried fruits are used in some breakfast cereals, and freeze-dried ice cream is an example of astronaut food. The main disadvantage of freeze-dried foods is that they are quite expensive due to the special equipment needed for the process.

When comparing freeze drying and spray drying, there is no gold standard about which one is better; it depends on the final application.

**Table 3:** Examples of the most common incorporation methods of bioactive ingredients within the edible food matrix and coatings.

Incorporation Method within Edible Matrix	Advantages	Disadvantages	Reference
Emulsions and nanoemulsions	- The possibility of incorporation of polar, non-polar, and amphiphilic compounds into the same delivery system - Different rheological ranges (from viscous liquids to plastic) - Direct use in “wet” state or drying to powders - Emulsions can be made entirely from food-grade ingredients (such as water, oil, surfactants, phospholipids, proteins, and polysaccharides) - Easy processing (mixing and homogenising)	- Susceptible to physical instability - Limited protection and controlled release due to small droplet size - Limited number of emulsifiers	[114]
Liposomes	- High bioavailability and absorption compared to other oral forms of supplementation - Increased intracellular delivery - Ability to deliver both hydrophilic and hydrophobic compounds simultaneously - Cost effective due to high bioavailability	- High industrial cost and scale-up problems - Poor stability under the complex environmental conditions - Potential difficulties in finding suitable food-grade substances - Manufacturing-related issues such as non-reproducibility from batch to batch, lack of effective sterilization methods	[115,116,117]
Solid lipid nanoparticles	- Increased stability and prolonged release - Slowing down the diffusion of pro-oxidants - Improvement of bioavailability	- Limited loading capacity for hydrophilic compounds - Gelation of lipid dispersions	[118]
Nanofibers	- Non-mechanical engineering → structural advantages: ultrafine structures, high porosity, high surface-to-volume ratio, tailored morphology - Nanofibrils can adsorb at the oil/water interface and form a coating around the oil droplets - Prolonged release time of the active ingredients - Non-thermal approach/protection against thermal degradation and possibility of encapsulation of thermosensitive compounds - High efficiency of incorporated bioactives - Reduced amount of organic solvents - Sustainability and environmentally-friendly	- Low productivity - So far, no data available on the long-term stability of compounds produced - Electrospinning is currently only used on a laboratory scale - Potential environmental and health risks of nanocomponents still quite unexplored	[102,119]
Inclusion complex	- Protection of lipophilic food ingredients from oxidation and degradation by light, heat - Improved thermal stability - Improved bioaccessibility - Improved water solubility of hydrophobic compounds - Ability to mask the bad taste of certain substances → reduction of organoleptic effects of volatile compounds - RH controlled release	- For polymers used in the food industry, the durability has not been studied so far - Polymer preparation usually does not follow the concept of green chemistry and new preparation methods should be developed	[120]
Complex coacervates	- Higher thermal degradation temperatures than their individual biopolymers - High payload - Process at low temperature - Reduced evaporation losses - Compatibility to control the release of active ingredients - Improvement of chemical stability of sensitive compounds	- The high cost of the particle isolation processe and the complexity of the technique should be considered	[121,122,123]

### 4.4. Release Mechanisms

The release of encapsulated ingredients can be site-specific, stage-specific, or triggered by changes in pH, temperature, irradiation, or osmotic shock [83]. The mechanism of release in the food industry is often through solvent-activated effects, such as melting, diffusion, degradation, or particle breakage [124]. For example, unencapsulated sweeteners in chewing gum are released at the onset of chewing, whereas encapsulated sweeteners are released slowly through the sheer action of chewing. The result is a more intense flavor as chewing time progresses. Vitamins are nutrients that are well known for their importance in the human body. However, they are often not properly absorbed by human bodies, resulting in few or no benefits. Bad chemical stability under various manufacturing and storage conditions and in the gastrointestinal tract limits their absorption and bioavailability. The authors of [125] provided a comprehensive literature review of the release profiles of vitamin C, an example of a water-soluble vitamin encapsulated by various processes. Recently, the authors of [126] showed that chitosan-modified cellulose nanocrystal formulations can control and accumulate the release of vitamin C in the upper stomach (pH 5), lower stomach (pH 2), duodenum (pH 7.4), and small intestine (pH 5) up to 8 h compared with a neutral control (pH 7.4). The release of fat-soluble vitamins, such as vitamin B, can be controlled by encapsulation in liposomal nanocarriers with significantly improved retention compared to unpackaged vitamin B1 under physiological conditions and in artificial gastric juice [95].

## 5. How to Evaluate the Activity of the Active Compound?

Due to the growing public awareness of the importance of a diet rich in bioactives, the scientific community has been challenged to determine their activity in order to obtain reliable data and evidence of their health-promoting effects. The bioavailability and bioactivity are limited by their stability primarily against the oxidative degradation during processing and by bio-accessibility from various encapsulated forms during the gastrointestinal digestion. Estimation of bioavailability in food can be done by in vitro methods and in vivo. In vitro methods provide knowledge on the digestion and absorption of simulated GI models, on possible interactions between compounds and carriers (encapsulation agents and food), the effects of medium conditions (pH, temperature, enzymes), and processing conditions among others. In vivo methods cover clinical trials and dosing the experimental humans or animals with several concentrations of the targeted bioactive to measure its antioxidant, anti-inflammatory, anticancer, or antimicrobial effect among others. The human organism is a complex system and several factors play an important role in the metabolism of bioactives. Therefore, there is an increasing interest in conducting in vivo studies in humans to better understand the fate and potential activity of the observed compounds. As different experimental approaches to characterize activity and availability exist, in this review the focus will be made on the bioactives with antioxidant properties. 

### 5.1. Evaluation of the Antioxidant Activity

In general, the antioxidant activity of bioactive compounds is attributed to their ability to neutralize the action of free radicals. The mechanism of their action may roughly rely on two different types of reactions: electron transfer (ET) or hydrogen atom transfer (HAT) [127] and, accordingly, the available in vitro methods for measuring antioxidant activity are based on one of these two reactions. ET assays measure the ability of the antioxidant to transfer an electron to reduce radicals, metals and carbonyl groups, whereas HAT assays measure the ability of the antioxidant to donate hydrogen and consequently quench free radicals [128]. The ET assays include trolox equivalent antioxidant capacity (TRAP), ferric ion reducing antioxidant parameter (FRAP), Folin-Ciocalteu’s phenol reagent reducing ability 2,2-Azinobis 3-ethylbenzthiazoline-6-sulfonic acid radical scavenging assay (ABTS+), and diphenyl-1-picrylhydrazyl (DPPH) copper (II) reduction capacity, whereas HAT assays include oxygen radical absorbance capacity (ORAC), total radical trapping antioxidant parameter (TRAP), inhibition of induced low-density lipoprotein, LDL, oxidation, total radical scavenging capacity assay (TOSCA), β-carotene bleaching, and chemiluminescent assay [129,130]. Although research has produced numerous in vitro assays to evaluate antioxidant activity, unfortunately there is no single method that can demonstrate the efficacy of the bioactive compound as an antioxidant. First, the most commonly used radicals in in vitro assays such as DPPH and ABTS are not relevant in biological systems. Those that are relevant are used in excessive concentrations, which again do not reflect biological conditions [131]. In addition, the literature review shows that it is difficult to compare the results of such assays between different laboratories, and the results between two different antioxidant assays are not comparable. All these observations have raised doubts about the reliability of these methods and brought their drawbacks and limitations into the spotlight. A step away from these limitations is offered by cellular assays that provide a better estimation of antioxidant activity at the cellular level [132]. These assays primarily include the haemolysis assay on red blood cells and the more widely used cellular antioxidant assay on human haepatocarcinoma cells, which provides a cost-effective solution between in vitro chemical assays and the most reliable in vivo studies. To best evaluate the antioxidant activity of bioactives, one should certainly resort to in vivo studies in animals or humans. However, these studies are expensive, lengthy, complex, and often require a rigorous regulatory process [133]. Considering all the aforementioned, the general practice to estimate antioxidant activity by using in vitro assays is currently to use multiple assays (at least three and preferably based on different reaction mechanisms), as well as a reference compound with demonstrated antioxidant activity. This type of analysis should allow the observation of the general potential of bioactive molecules to act as antioxidants. Apart from these methodological limitations, another major problem related to bioactive compounds as potential antioxidants is their behavior and fate in a complex system such as the human organism.

Chemical and cellular assays can reveal the potential for antioxidant activity, but the greatest concern is the bioaccessibility and bioavailability of bioactives in the human organism. Bioaccessibility is defined as the amount of bioactive compound(s) that can be released from the food matrix into the gastrointestinal tract, while bioavailability determines the proportion of the compound that can be ingested and utilized [134]. Because dietary polyphenols comprise different classes of polyphenols, their bioaccessibility and bioavailability depend largely on their structure (degree of polymerization, type and number of functional groups, etc.) as well as on external factors such as interaction with the food matrix and the characteristics of the gut microbiota [135,136]. It is well known that most dietary bioactives, especially polyphenols, have low bioavailability. Estimates show that nearly 90% to 95% of polyphenols reach the colon in an intact form [137]. Currently, microencapsulation techniques are considered an effective tool to increase bioaccessibility and bioavailability of bioactives by protecting the active ingredient from degradation in the gastrointestinal system, increasing the solubility of the active ingredient, and improving its penetration through the intestinal barrier [138]. 

### 5.2. In Vitro vs. In Vivo

The methodology for assessing bioavailability includes in vitro and in vivo testing. In vitro tests are based on the simulation of gastrointestinal digestion and possibly the addition of digestate to human Caco-2 cells [134]. As with in vitro antioxidant assays, these assays only provide insight into the potential utilization and digestibility of antioxidants. Nevertheless, even in vivo studies lead to different conclusions because most antioxidants undergo extensive metabolic changes, and it is difficult to detect the parent compound and prove its activity. Additionally, there is evidence of interaction with the human gut microbiota and microbial enzymes that cause further transformation [139]. Therefore, it is generally believed that possible beneficial effects of antioxidants in the organism can be attributed to their metabolites [140,141], as some metabolites show even better absorption and efficacy than the native molecule [138]. An example of this is the so-called “resveratrol paradox”, where the low bioavailability and high bioactivity is attributed to the metabolites, as more than 90% of orally administered resveratrol is excreted via urine and feces [140]. On the other hand, the gut microbiota is the fingerprint of the human organism and differs from individual to individual. Therefore, it could be the most important key to explore the final antioxidant and health-promoting effects of dietary bioactives. Thus, the scientific community still needs to focus its research on a better understanding of the final antioxidant effect of bioactive compounds on the human organism in order to develop comparable and reliable tools and methods for their measurement. Currently, with the available methodology, the general recommendation is that, when using in vitro methodology for an antioxidant’s assessment, one should apply several assays with different reaction mechanisms coupled with a determination of bioavailability or, preferably, apply the in vivo methodology. The gold standard is the clinical trial, which in the age of global awareness toward the use of natural antioxidants should be encouraged, as they are the only reliable tool for proving the bioactives’ effectiveness on oxidative stress and related health conditions. Several plant bioactives have been subject to clinical trials, with *Ginkgo biloba* L. extract being one of the most extensively studied for its effect on the improvement of cognitive function [142,143,144]. The available results show opposite conclusions, reporting both positive and negative outcomes. The reason why even clinical trials may fail to provide the reliable evidence of the effectiveness of bioactives is mainly due to the duration of the study, administration dose, population criteria, and characteristics and effect outcome measures or applied oxidative stress markers [145,146]. Therefore, a careful approach and future developments are needed for the evidence-based measurement of the activity and related beneficial effects of bioactives. 

## 6. Effect on Consumers

Although the effect of plant bioactives on health has been demonstrated in many examples, it seems to correlate more with a diet rich in fruits and vegetables, but rarely with the intake of individual active ingredients, which may even have a negative effect [147]. For example, carotenes are well-known antioxidants [147,148,149], but supplementation can result in adverse effects for some specific populations [150]. In addition, plants rich in lycopene may have several beneficial effects, notably reducing cardiovascular disease [151] or prostate cancer, especially advanced or fatal cancers, whereas lycopene supplementation has far less impact [152,153]. This difference might be due to interactions between compounds, as has been shown between carotene and lycopene [47] but also to a difference in bioactivity of the mixture compared to the single molecule. These differences could also be due to the fact that vitamins or antioxidants present in food matrices are more stable and better able to withstand the extreme conditions of the digestive tract (acidic pH in the stomach, basic pH in the intestine) when naturally embedded in food matrices (plant or animal cells) rather than isolated in pure form. The digestive stability of compounds such as carotenoids or certain vitamins can reach 90% in spinach, the orange-fleshed sweet potato, oranges, broccoli, or salad greens [154,155,156]. This improved digestive stability is even observed in liquid foods such as carrot juice. The location of the compounds within the cells and the richness of the food in other dietary antioxidants appear to be critical [157]. 

It is therefore very important to scientifically evaluate the effects of foods or food components on consumer health before saying anything about a possible effect. National or supranational agencies have the task of checking whether health claims are based on scientific evidence. Health claims refer to the relationship between a food or food component and health. In the European Union, there are several types of health claims: health function claims, risk reduction claims, and child development claims. Because these claims are based on scientific evidence, it is often difficult to obtain them, leading to bitterness among companies that have been denied by authorities. To be successful, it is important to adequately define the study involving a food or ingredient and the claimed effect and beneficial physiological effect, and to provide sufficient data from relevant human studies to support the claim. Reports of human intervention and observational studies are the key documents considered by the agencies in their review. However, to clarify scientific issues, they may also consider other data analyses such as meta-analyses or reviews, as well as animal or in vitro studies. These aspects are explained in the review [158] and discussed for carotenes [159]. 

These examples show that novel foods consisting of new sources of carotene may be acceptable in the EU, but it is rather difficult to obtain health claims in this field. Furthermore, health claims of dietary supplements refer to the healthy population (including specific groups), but these products cannot be used as medicines. For example, provitamin A carotenoids have been shown to have a very positive effect in increasing serum vitamin A levels in poor children in rural Vietnam, whereas dietary supplementation trials were stopped prematurely because of a higher risk of lung cancer in smokers [160].

In many Western countries, dietary policy recommends a varied diet based on all food groups and provides consumers with guidance on ideal frequencies and portions. This dietary diversity strategy offers no magic solutions and encourages a balanced diet throughout the week [161]. Recently, a decision support model has been developed that predicts how dietary choices affect life expectancy [162]. Dietary change can provide significant health and life expectancy benefits for people of all ages, with the effect of vegetables and whole grains being most impressive. In low- and middle-income countries, governments are promoting food fortification, i.e., the addition of an active ingredient to a common consumer product such as fish sauce, to stay in the Vietnamese context. Poor populations who lack physical and economic access to a healthy diet are targets of “base of the pyramid” strategies (i.e., for many people at the bottom of the income pyramid), thus creating a nutrition market that is expected to yield quick nutritional results. In these contexts, regulation by some scientific agencies is often lax, monitoring is infrequent, and consumers are confused by non-scientific communication, which can lead to overconsumption of bioactives by the high-income population. Some examples of the dissemination of non-scientific data were cited in a review on the gac fruit, which showed how a false concentration of ascorbic acid was attributed to this fruit following a recipe mixing three superfruits [24].

## 7. Conclusions

This review presents the issues related to the booming production and use of bioactives and discusses the technologies that can help to manage their extraction, encapsulation, delivery, and evaluation. It gives a scientific perspective on how to respond to the increasing demand for health-promoting products. It is clear that, despite the existence of truly bioactive products, there are still many challenges that need to be overcome. From a marketing perspective, bioactives often benefit from a strong image among consumers, which unfortunately does not always match scientific reality. Addressing the quality challenge will help develop a scientific assessment of the bioactive’s effects that could lead to more claims, despite the complex nutritional interactions. Of course, a better understanding of the mechanisms of action of active ingredients will also help in formulation and delivery, as well as in obtaining claims and finding new bioactive ingredients. However, the boom in this area raises other issues besides quality such as sustainability and safety of production [8]. The impact of overexploitation of resources on local populations is also an issue, despite the Nagoya Protocol on Access to Genetic Resources and the Fair and Equitable Sharing of Benefits Arising from their Utilization implementing the obligations of the Convention on Biological Diversity. These groups aim to protect local populations by sharing benefits, despite the appetite of big industrial groups, and the existence of illegal trafficking, the presence of which often results in complete changes to the living conditions of traditional communities. From a technical point of view, considering the diversity of raw materials, of chemical structures and physicochemical properties, of effects and of targeting tissues, a huge diversity of ways of extraction, of delivery, of evaluation are needed in conditions that can be upscaled to the industrial scale without pollution. Although technologies based on new principles without toxic solvents have been developed, many technical progresses are still needed. Finally, the use and promotion of products providing health benefits have to be tied to clear and science-based standards of health protection adapted to the population particularity.

## Figures and Tables

**Table 1:** Comparison of novel and conventional extraction methods of polyphenols from plants.

Novel Extraction Method	Conventional Extraction	Plant	Extraction Parameters	Solvent Type	Comparison of Novel vs. Conventional Extraction	Reference
MAE	Soxhlet	Daisy (*Vernonia amygdalina* L.)	MAE: 5–15 min, power 400–600 W, 90–110 °C Soxhlet: 8 h, 100 °C	Water	MAE vs. Soxhlet: 38% higher e.y. and 48 times shorter treatment period	[58]
UAE	Water bath shaking technique (WBST)	Sage (*Salvia officinalis* L.)	UAE: 100% amplitude, 400 W water bath: 30 min, 60 °C	Water	UAE vs. WBST: 23% higher e.y. in UAE 3 times shorter treatment	[59]
				30% ethanol	UAE vs. WBST: 6% higher e.y. in UAE, 3 times shorter treatment	
				30% acetone	UAE vs. WBST: 19% lower e.y. in UAE, 3 times shorter treatment	
ASE and SFE	Soxhlet, Shaking sample/methanol	Peppermint (*Mentha piperita* L.)	ASE: solvent: methanol; 35 °C SFE: CO_2_ flow 40 g/min	3% (*v*/*v*) methanol	No benefits from SFE ASE vs. Soxhlet: 44% higher e.y. ASE vs. Sample shaking: 92% higher e.y.	[60]
		Oregano (*Origanum vulgare* L.)			ASE vs. Soxhlet: 22% higher e.y. ASE vs. Sample shaking: 72% higher e.y.	
		Rosemary (*Rosmarinus officinalis* L.)			ASE vs. Soxhlet: 22% higher e.y.ASE vs. Sample shaking: 14% higher e.y.	
		Thyme (*Thymus vulgaris* L.)			ASE vs. Soxhlet: 40% higher e.y. ASE vs. Sample shaking: 64% lower e.y.	
EAE, MAE and MEAE	Solvent extraction	Olive pomace from *Biancolilla*, *Cerasuola*, and *Nocellara cultivars*	MAE: 17 min, 600 W, 35–60 °C EAE: cellulase, pectinase, and tannase; 50 °C, 120 rpm for 2 h	Ethanol/water	MAE vs. solvent extraction: 11% higher e.y. in 7× shorter treatment EAE vs. solvent extraction: 55% higher e.y., best result for cellulase MEAE: improvements compared to EAE only for cellulase (95%)	[61]
PEF	Solvent extraction	Greek mountain tea (*Sideritis scardica* Griseb.), saffron crocus (*Crocus sativus* L.)*,* grape vine (*Vitis vinifera* L.)	PEF: 20 min, 10 μs, field intensity 1.2–2.0 kV/cm	Water	PEF vs. solvent extraction: 49% higher e.y.	[62]
SFE	Soxhlet extraction	Tomato peel and seed, by-products	SC-CO_2_: 20 min, CO_2_ flow rate at 1 L/min; 40–80 °C, 30–50 MPa	Hexane	30% shorter extraction time for SFE than Soxhlet extraction	[63]

MAE, microwave assisted extraction; UAE, ultrasound assisted extraction; ASE, accelerated solvent extraction; SFE, supercritical fluid extraction; EAE, enzyme assisted extraction; MEAE, microwave assisted extraction coupled with enzyme assisted extraction; PEF, pulsed electric field; e.y., extraction yield.

**Table 2:** Comparison of novel green extraction methods used for the extraction of flavonoids from grape seeds.

Method	Grape Variety/Sample Type	Processing Conditions	Total Flavanoids Content	Major Compounds Determined	Reference
MAE	*Vitis vinifera* L., “Napoleon” variety, skin and seeds	100 °C; solvents: methanol, ethanol, acetone, and water; 100–500 W; ratio sample to solvent 10–50 mg mL^−1^; 5–20 min; magnetic stirring: 0–100%	75.9 mg/100 g + 2.2 mg (caffeic acid equivalent)/100 g + 6.1 mg (rutin equivalent)/100 g	phenolic acids: caftaric acid flavanols: catechin, epicatechin flavanol glycosides: dihydrokaempferol-glycoside, quercetin, quercetin-3-*O*-glucoside, kaempferol-3-*O*-glucoside	[64]
UAE	*Vitis vinifera* L., Syrah variety, grape skin residue	80% ethanol; 55 ± 5 KHz ultrasound; 190 rpm; 25 °C–60 °C; 10–30 min	9.8–40.0 mg quercetin equivalent/100 g	phenolic acids: gallic acid, caffeic acid, caftaric acid flavanols: catechin, procyanidin B1, procyanidin B2 flavanol glycosides: kaempferol-3-*O*-glucoside, quercetin-*β*-*D*-glucoside, isorhamnetin-3-*O*-glucoside-chloride, myricetin, rutin anthocyanins: malvidin-3-*O*-glucoside-chloride, cyanidin-3-*O*-glucoside-chloride, pelargonidin-3-glucoside-chloride, delfinidine-3-*O*-glucoside, peonidine-3-*O*-glucoside stilbenes: cis-reservatril, trans-reservatrol, viniferin	[65]
ASE	*Vitis vinifera* L., cv. Cabernet Sauvignon, wet and dry grape pomace	45 °C–140 °C; solvent: 70% ethanol/water	∼1000 mg GAE/100 g at 140 °C for wet pomace and 600 mg GAE/100 g at 80 °C for dry pomace	phenolic acids: gallic acid, protocatechuic acid flavanols: gallocatechin, catechin, prodelphinidin B3, epicatechin	[66]
EAE	*Vitis vinifera*, cv. Regen, grape skin	45 °C; 3 h; pH 2.0, enzyme types: EX-V*, HC*, ER*, ECP*, enzyme dosage of 10.52 mg/g	4581.7 mg/100 g (with EX-V*) 4467.7 mg/100 g (with HC*)4358.8 mg/100 g (with ER*)4316.9 mg/100 g (with ECP*)	flavanols: galocatechin, procyanidin B1, epigallocatechin, catechin, procyanidin B2, epicatechin flavanol glycosides: myricetin-3-*O*-glucoside, rutin, quercetin-3-*O*-glucuronide, quercetin-3-*O*-glucoside, kaempferol-3-*O*-glucuronide, isorhamnetin-3-*O*-glucoside anthocyanins: delphinidin-3,5-*O*-diglucoside, cyanidin-3,5-*O*-diglucoside, delphinidin-3-*O*-glucoside, peonidin-3,5-*O*-diglucoside, malvidin-3,5-*O*-diglucoside, cyanidin-3-*O*-glucoside, peonidin-3-*O*-glucoside, malvidin-3-*O*-glucoside	[67]
SLE		70% aqueous ethanol containing 1% formic acid for one day in the dark; 40 °C	4462.2 mg/100 g		

MAE, microwave assisted extraction; UAE, ultrasound assisted extraction; ASE, accelerated solvent extraction; SLE, solid-liquid extraction; EAE, enzyme assisted extraction; GAE, Gallic acid equivalent; EX-V*, enzyme type: Lallzyme EX-V; HC*-enzyme type: Lallzyme HC; ER*-enzyme type: Endozym rouge; ECP*-enzyme type: Endozym contact pelliculaire; * as given in [68].

## Data Availability

The data presented in this study are available in review.
